# In vitro and in vivo anti-cancer effects of hibernating common carp (*Cyprinus carpio*) plasma on metastatic triple-negative breast cancer

**DOI:** 10.1038/s41598-022-06368-4

**Published:** 2022-02-21

**Authors:** Mojtaba Golpich, Elham Amini, Amirhosein Kefayat, Mehrafarin Fesharaki, Jamal Moshtaghian

**Affiliations:** 1 Department of Plant and Animal Biology, Faculty of Biological Science and Technology, University of Isfahan, 81746-73441 Isfahan, Iran; 2 Department of Cell and Molecular Biology and Microbiology, Faculty of Biological Science and Technology, University of Isfahan, Isfahan, Iran; 3 Department of Oncology, Isfahan University of Medical Sciences, Isfahan, Iran; 4 Department of Cell Sciences Research Center Medical Sciences, School of Medicine, Isfahan University of Medical Sciences, Isfahan, Iran

**Keywords:** Breast cancer, Cancer prevention, Cancer therapy, Metastasis, Cell death, Cell division, Cell growth, Cell migration

## Abstract

Uncontrollable proliferation is a hallmark of cancer cells. Cell proliferation and migration are significantly depressed during hibernation state. Many studies believe some factors in the plasma of hibernating animals cause these effects. This study aimed to assess the anti-cancer effects of hibernating common carp (*Cyprinus carpio*) plasma on 4T1 cancer cells in vitro and in vivo. The effect of hibernating plasma on cell viability, morphology, migration, apoptosis rate, and cell cycle distribution of 4T1 cells was investigated in vitro and in vivo. Hibernating plasma at a concentration of 16 mg/ml significantly reduced the viability of 4T1 cancer cells, without any toxicity on L929 normal fibroblast cells. It could change the morphology of cancer cells, induced apoptosis and cell cycle arrest at the G2/M phase, and inhibited migration. Furthermore, intratumoral injection of hibernating plasma (200 µl, 16 mg/ml) in the tumor-bearing mice caused a significant inhibition of 4T1 breast tumors volume (46.9%) and weight (58.8%) compared with controls. A significant decrease in the number of metastatic colonies at the lungs (80%) and liver (52.8%) of hibernating plasma-treated animals was detected which increased the survival time (21.9%) compared to the control groups. Immunohistochemical analysis revealed a considerable reduction in the Ki-67-positive cells in the tumor section of the hibernating plasma-treated animals compared with controls. Taken together, the SDS-PAGE and mass spectrometry analysis indicated the alpha-2-macroglobulin level in the hibernating fish plasma was significantly increased. It could exert an anti-cancer effect on breast cancer cells and suggested as a novel cancer treatment strategy.

## Introduction

Cancer is a major public and economic health issue, and breast cancer with two million newly diagnosed cases each year is the most common malignancy and the leading cause of cancer deaths in women worldwide^1^. The most invasive subtype of breast cancer is triple-negative breast cancer (TNBC)^2^. Current therapeutic strategies for TNBC patients are unsatisfying and often associated with a high rate of recurrence^3^. Therefore, there is an urgent need to identify novel treatments for this subtype of breast cancer. Because the main hallmark of cancer cells is uncontrollable proliferation^4^, novel agents with anti-proliferative effects can be used as potential cancer treatments.

Hibernation or torpor is a state of minimal activity and metabolic depression. During this state, different organisms enter a specific hypometabolic and dormant state to survive harsh environmental conditions including reduced food supply and low ambient temperature. In this state, organisms experience significant changes including a considerable alteration in gene expression patterns of different organs, a dramatic metabolic suppression, a drop of core body temperature to the ambient temperatures, a decrease in the heart and respiratory rates, and a shutting down of the digestive tract^5–7^. Generally, most cellular activities are dramatically suppressed during hibernation for saving energy. Mitosis is one of the most energy-demanding activities for cells. Therefore, it is not surprising that the rate of cell proliferation while organisms are in hibernation or torpid state is drastically decreased, which is consistent with a profound reduction in the synthesis of DNA, RNA, and proteins^8–16^. The gastrointestinal system has been the most important target organ for the investigation of the hibernation effect on cell proliferation due to the high rate of enterocyte division^14,17^. DNA synthesis in epithelial cells of the small intestine is reduced by about 4% of the normal rate during the torpid state. Enterocytes are stopped at the G2 or late S phase of the cell cycle and their migration from the proliferation zone of crypts to the villus tips is almost arrested^18,19^.

During hibernation or deep torpor, mitotic activity can be completely ceased^19^, however, the mechanism that prevents cells from entering mitosis in this condition is poorly understood. There are some hypotheses to explain the reduction of mitosis during hibernation including decreased enzymatic activity due to temperature decline or decreased production of growth factors^13^. Additionally, many studies have indicated that there are several factors in the plasma that can induce hibernation and suppress mitosis. Interestingly, it has been shown that transferring the plasma of hibernating animals to other animals of the same or even different species causes hibernation^20–23^. Moreover, the proliferation rate of cultured murine splenocytes incubated with the plasma of hibernating 13-lined ground squirrels is significantly decreased in a dose-dependent manner^24^. Furthermore, collected plasma from hibernating American bullfrog (*Lithobates catesbeianus*) exhibited anti-proliferative effects on a human monocytic cell line (THP-1) derived from a patient with acute monocytic leukemia^13^. Taking together, the plasma of hibernating animals, which has shown a high ability to induce the cell cycle arrest and anti-proliferative effects on cells can be considered as a potential target for cancer therapy.

The main aim of the present study is to investigate the anti-proliferative and anti-metastatic effects of hibernating common carp (*Cyprinus carpio*) plasma on breast cancer cells in vitro and in vivo. In this study, we used the 4T1 murine mammary tumor cell line, which is one of the most invasive and tumorigenic models of triple-negative breast cancer in Balb/c mice. To the best of our knowledge, this is the first study to evaluate the effect of hibernating animal plasma on the proliferation, morphology, apoptosis rate, cell cycle, migration, tumor growth, and metastasis of breast cancer. In our study, common carp (*Cyprinus carpio*) was used as an ectothermic organism to supply hibernating plasma. It is one of the most globally distributed and cheapest farmed fish that enters a hibernation state with decreasing temperature^25,26^.

## Results

### Anti-proliferative effect of hibernating plasma on cancer cells

**Figure 1:**
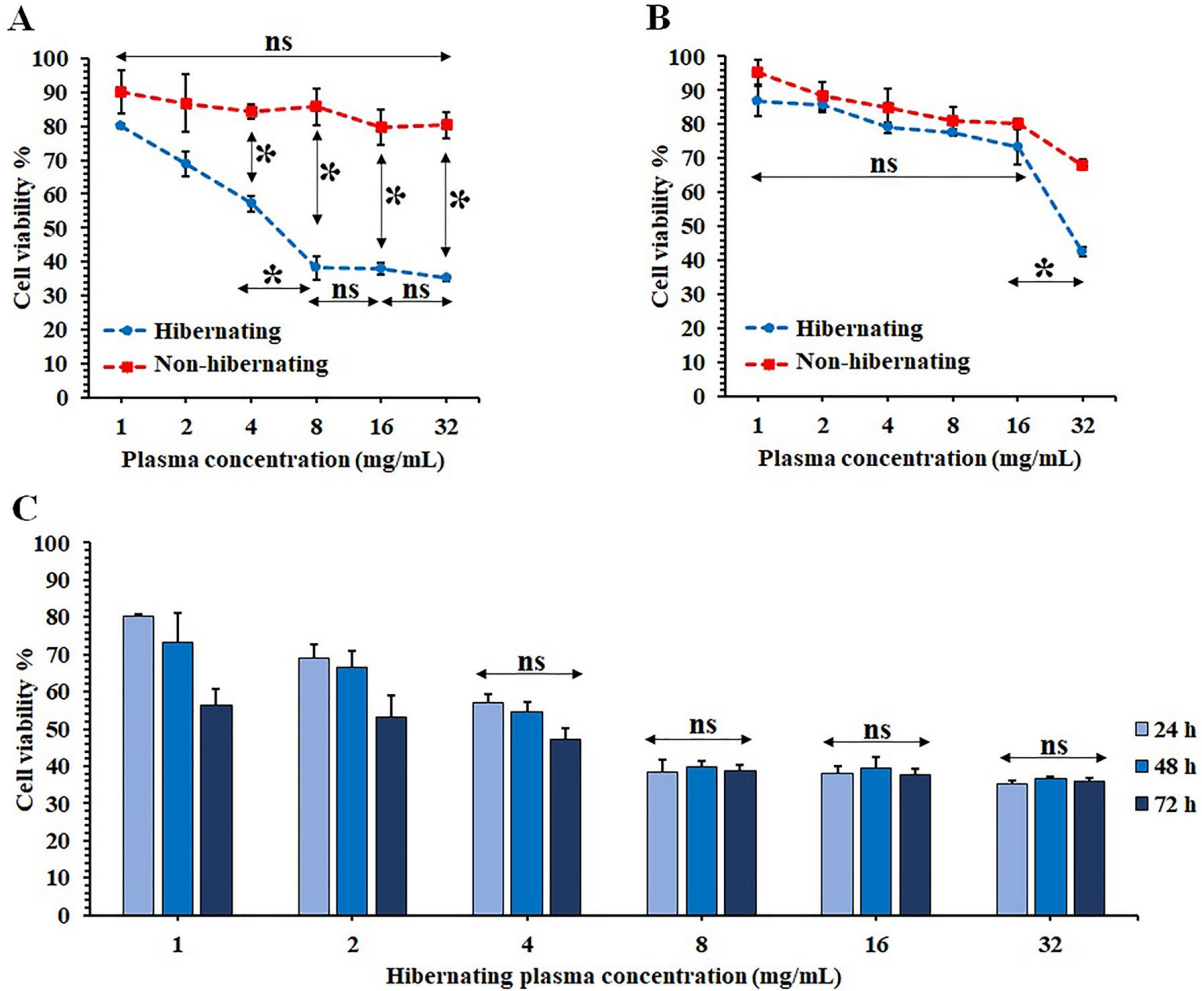
Effects of hibernating plasma on 4T1 breast cancer and L929 normal cell viability according to MTT assay. Comparison of different concentrations (1, 2, 4, 8, 16, and 32 mg/ml) of hibernating and non-hibernating plasma effects on the viability of (**A**) 4T1 breast cancer and (**B**) L929 normal fibroblast cells after 24 h incubation. (**C**) Comparison of anti-proliferative effect of different concentrations (1, 2, 4, 8, 16, and 32 mg/ml) of hibernating plasma on 4T1 cancer cells after different incubation times (24, 48, and 72 h). (ns: non-significant. *: *P* < 0.05).

The 4T1 murine triple-negative mammary cancer and L929 normal murine fibroblast cell lines were treated with different concentrations (1, 2, 4, 8, 16, 32 mg/ml) of hibernating and non-hibernating common carp plasma. Although non-hibernating common carp plasma did not exhibit any significant anti-proliferative effect on 4T1 cells, hibernating plasma significantly (*P* < 0.05) decreased cancer cell viability at concentrations of 4, 8, 16, and 32 mg/ml (Fig. 1A). Additionally, hibernating and non-hibernating plasma did not have significant toxicity on normal cells at concentrations of 1, 2, 4, 8, and 16 mg/ml. However, 32 mg/ml of hibernating plasma could significantly decrease L929 cell viability (Fig. 1B). Thus, 24-h incubation with 4, 8, and 16 mg/ml of hibernating plasma caused significant anti-proliferative effects on 4T1 breast cancer cells without considerable toxicity on normal cells. At the next step, 4T1 cells were incubated with the same set of concentrations (1, 2, 4, 8, 16, and 32 mg/ml) of hibernating plasma for 24, 48, and 72 h to obtain the best incubation time to achieve the highest anti-proliferative effect of hibernating plasma (Fig. 1C). It was shown that increasing the incubation time to 48 or 72 h did not significantly increase the anti-proliferative effect of hibernating plasma at concentrations that exhibited the highest anti-proliferative effects after 24-h incubation (4, 8, 16, and 32 mg/ml). Taking everything into consideration, the highest concentration of hibernating common carp plasma with the most anti-proliferative effects on cancer cells and least toxicity for normal fibroblast cells (16 mg/ml for 24 h) was selected as the most optimal treatment regimen and duration for further in vitro experiments.

### Effect of hibernating plasma on the morphology of cancer cells and normal cells

**Figure 2:**
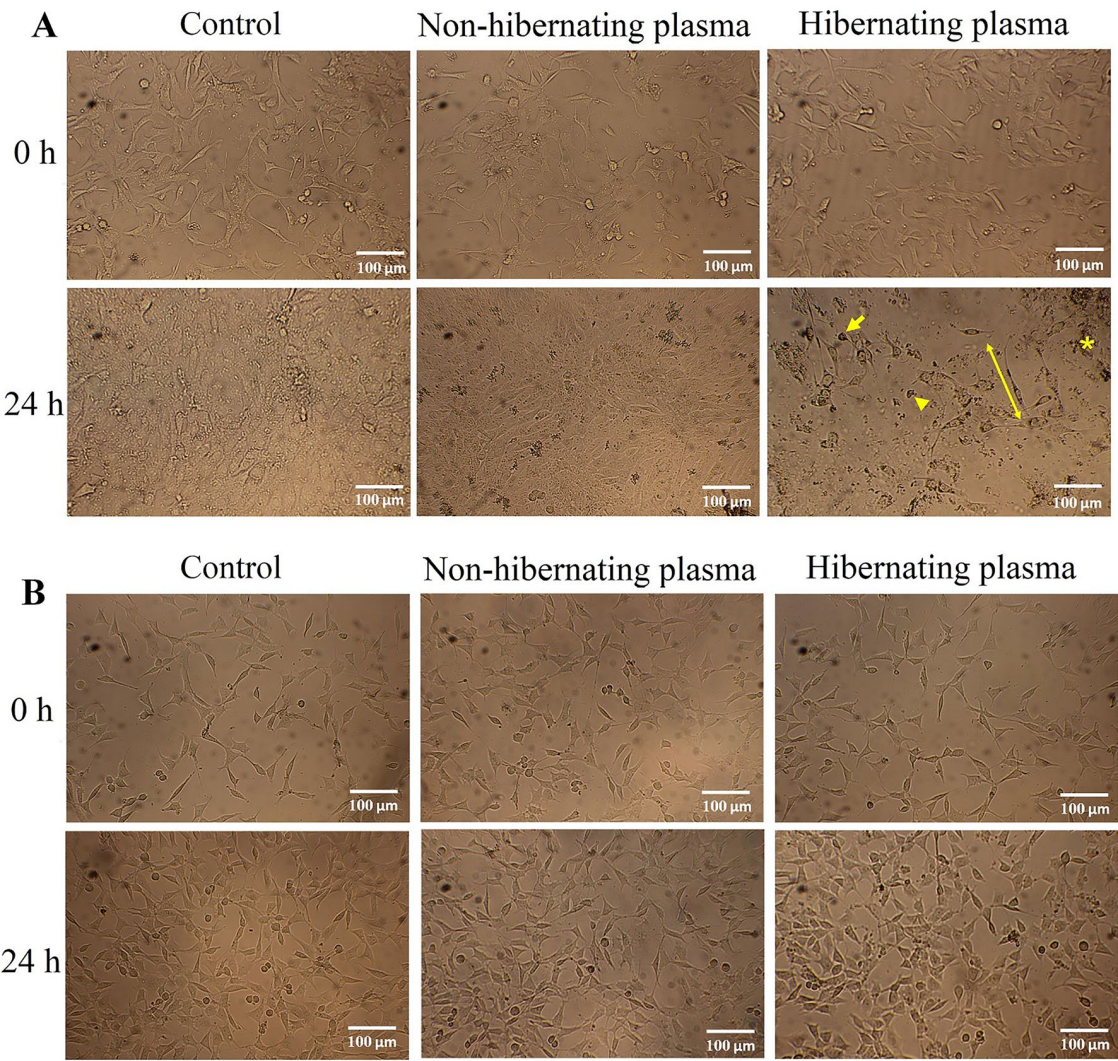
Effects of hibernating plasma on the morphology of 4T1 breast cancer and L929 normal cells. (**A**) 4T1 and (**B**) L929 cells photographs were obtained using a digital light microscope before (0 h) and 24 h after treatment with non-hibernating plasma (16 mg/ml) and hibernating plasma (16 mg/ml). The control cells were incubated with standard culture media. Arrow: rounded cell. Arrowhead: cell with irregular shape. Star: cell debris. Two-head arrow: cell with elongated cellular extensions.

To evaluate the effect of hibernating plasma on the morphology of cancerous and normal cell lines, 4T1 and L929 cells were treated with the selected hibernating plasma concentration (16 mg/ml for 24 h). Treatment with 16 mg/ml hibernating plasma for 24 h altered the morphology of cancer cells. A significant irregularity in the shape and size of 4T1 cancer cells was observed after hibernating plasma treatment compared with the non-hibernating plasma-treated and control cells. Most of the 4T1 cells became rounder and smaller. Moreover, a significant decrease in the cell number and confluence was observed after hibernating plasma treatment in comparison with the non-hibernating plasma-treated and control cells (Fig. 2A). In contrast, the same concentration of hibernating plasma (16 mg/ml for 24 h) did not affect the morphology and cell number of L929 cells (Fig. 2B).

### Pro-apoptotic effect of hibernating plasma on cancer cells

**Figure 3:**
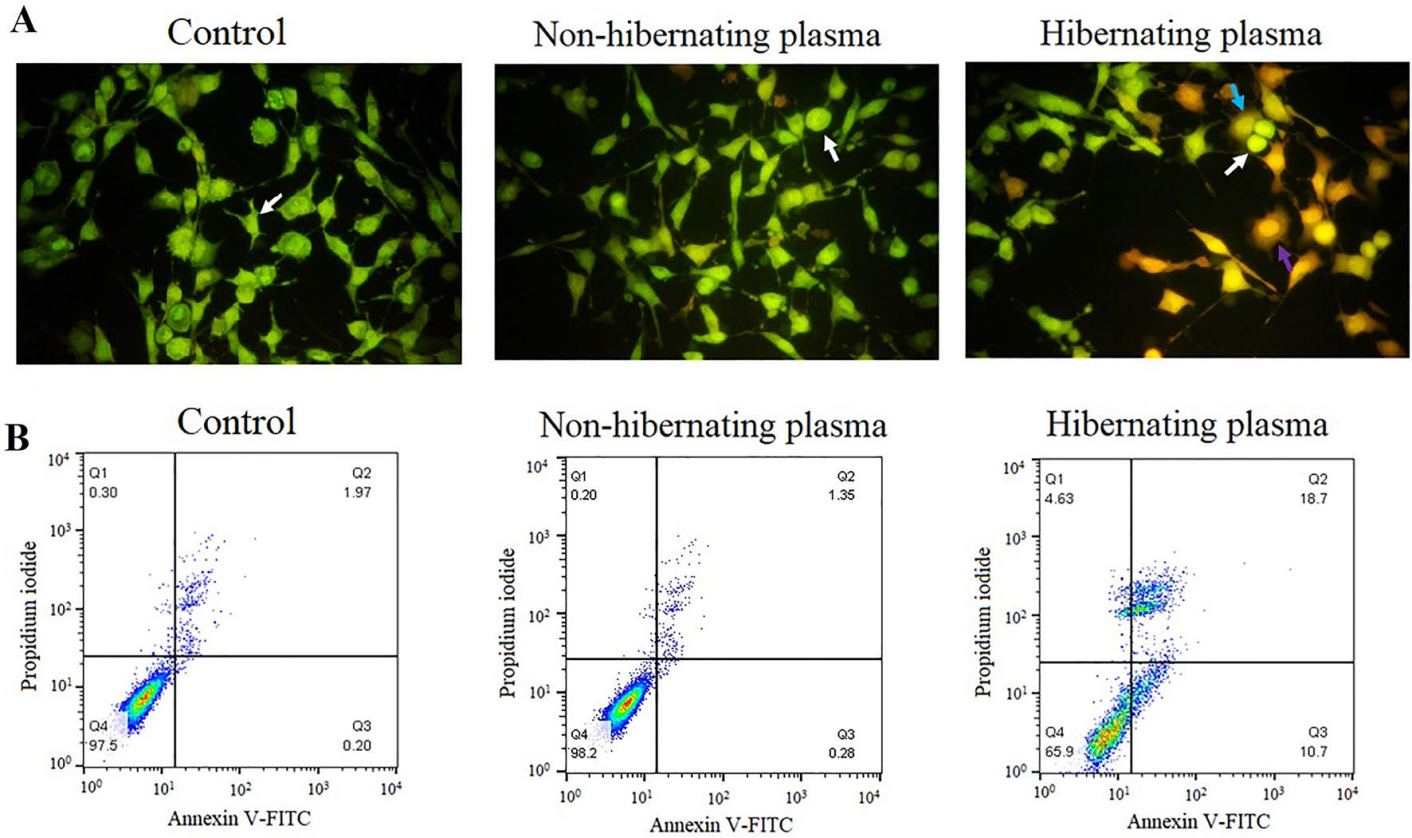
Evaluation of the ability to induce apoptosis in cancer cells by hibernating plasma according to the AO/EB double staining using a fluorescent microscope and the Annexin V-FITC/PI staining using flow cytometry. (**A**) The AO/EB double-stained 4T1 cancer cells after 24 h incubation with non-hibernating plasma (16 mg/ml) and hibernating plasma (16 mg/ml) under a fluorescent microscope (Magnification × 200). The control cells were incubated with standard culture media. Viable cells are stained uniformly pale-green (white arrow); Early apoptotic cells are stained bright-green to yellow (blue arrow); Late apoptotic cells are stained yellow-orange or red color (purple arrow); Necrotic cells can be detected as bright orange-red cells. (**B**) Quantification of hibernating plasma (16 mg/ml for 24 h)*-*induced apoptosis in 4T1 breast cancer cells by the Annexin V-FITC/PI staining using flow cytometry in comparison with non-hibernating (16 mg/ml for 24 h) and control. In each four-quadrant diagram, the lower left quadrant (Q4), lower right quadrant (Q3), upper right quadrant (Q2), and upper left quadrant (Q1) represent viable, early apoptotic, late apoptotic, and necrotic cells, respectively.

According to the AO/EB double staining assay, treatment with 16 mg/ml hibernating plasma for 24 h induced apoptosis in 4T1 cancer cells which was not observed in the non-hibernating plasma-treated and control cells (Fig. 3A). A considerable number of 4T1 cells treated with hibernating plasma exhibited apoptotic appearance. However, the control and non-hibernating plasma-treated cancer cells were completely viable and uniformly pale-green. For more quantitative analysis, the Annexin V-FITC/PI-stained cells were analyzed using flow cytometry (Fig. 3B). The viable (Annexin V-FITC^-^/PI^-^), early apoptotic (Annexin V-FITC^+^/PI^-^), late apoptotic (Annexin V-FITC^+^/PI^+^), and necrotic (Annexin V-FITC^-^/PI^+^) cells were quantitatively determined by this method (Table 1). Treatment with hibernating plasma significantly (*P* < 0.05) increased the percentage of total apoptotic (early and late apoptotic) cells, whereas, treatment with non-hibernating plasma showed no pro-apoptotic effect. The results were consistent with our observations in the AO/EB double staining experiment. Our findings indicated that hibernating common carp plasma induced apoptosis in 4T1 breast cancer cells.

**Table 1:** The mean percentage of viable cells, early apoptotic cells, late apoptotic cells, and necrotic cells after 24 h incubation with standard culture media (control), non-hibernating plasma (16 mg/ml), and hibernating plasma (16 mg/ml).

Phase	Control	Non-hibernating plasma	Hibernating plasma
Viable cells	96.6 ± 0.9% ^a^	96.2 ± 2% ^a^	60.4 ± 5.4% ^b^
Early apoptosis	0.6 ± 0.4% ^a^	1.2 ± 0.7% ^a^	13.8 ± 3.1% ^b^
Late apoptosis	1.7 ± 0.1% ^a^	1.8 ± 0.2% ^a^	20.3 ± 1.6% ^b^
Necrosis	1 ± 0.6% ^a^	0.7 ± 0.1% ^a^	5.3 ± 0.6% ^b^

Values represent mean ± standard deviation of three different experiments. In each row, levels are not connected by the same letters are significantly different (*P* < 0.05).

### Inductive effect of hibernating plasma on the cell cycle arrest at the G2/M phase in cancer cells

**Figure 4:**
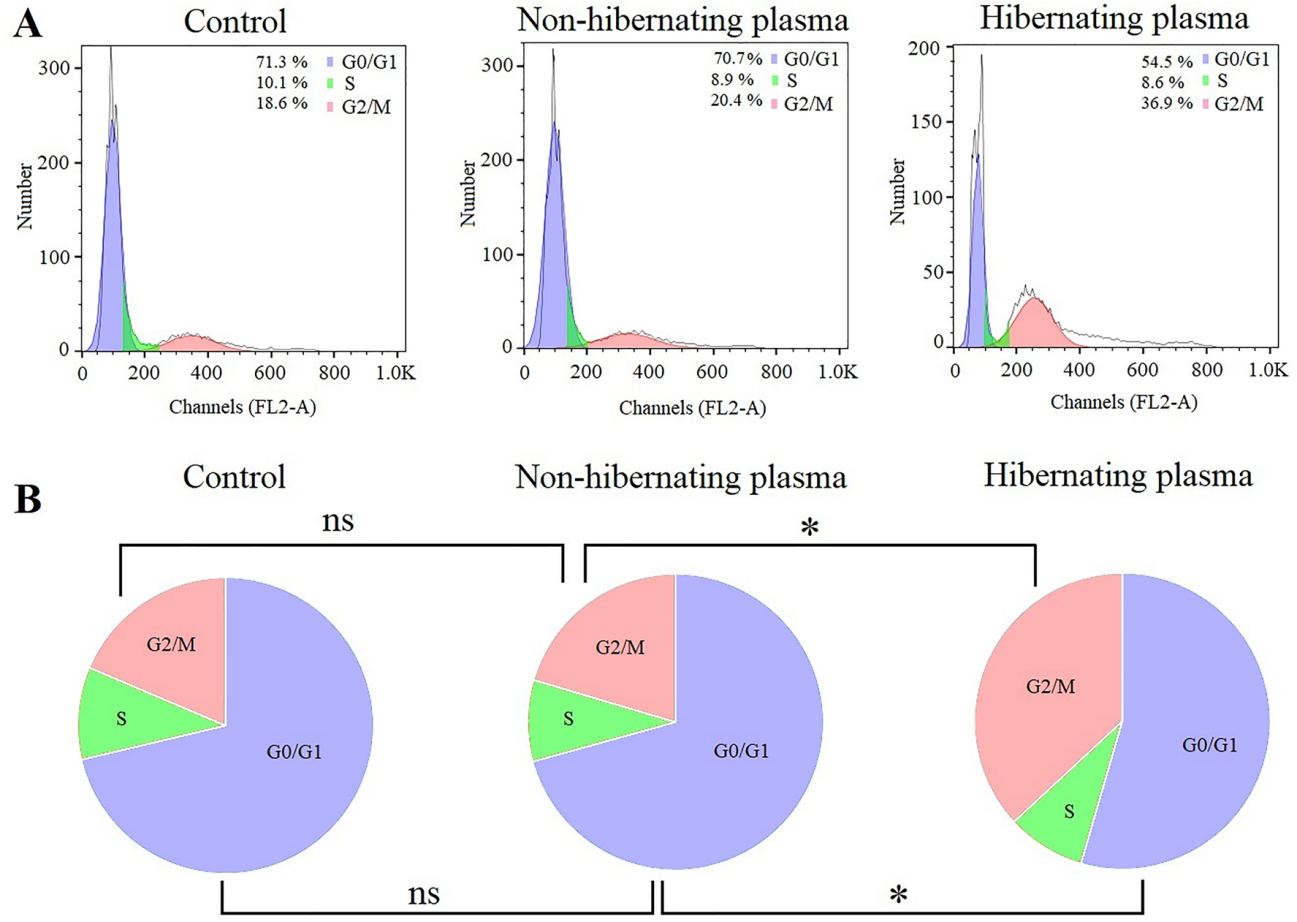
Evaluation of hibernating plasma effect on the cell cycle in cancer cells. (**A**) Representative flow cytometry cell cycle histogram of control, non-hibernating plasma (16 mg/ml, 24 h), and hibernating plasma (16 mg/ml, 24 h)-treated cells. (**B**) Pie charts of cancer cell distribution at different phases of the cell cycle in different groups. (*ns* non-significant. *: *P* < 0.05).

To assess the effect of hibernating common carp plasma on the proliferation of breast cancesr cells, the cell cycle alterations in 4T1 cells treated with hibernating plasma and non-hibernating plasma (after 24 h) were examined using flow cytometry. As Fig. 4 illustrates, the number of cancer cells at the G2/M phase was significantly (*P* < 0.05) higher in the hibernating plasma-treated wells (34.7 ± 6.3%) compared with the non-hibernating plasma (18.5 ± 5.5%) and control (18.3 ± 3.9%) groups. Besides, a significant (*P* < 0.05) decrease in the percentage of cancer cells at the G0/G1 phase was detected after treatment with hibernating plasma (54.2 ± 4.8%) in comparison with other groups (non-hibernating: 73.1 ± 6.4%, control: 71 ± 5.6%). There was no statistically significant (*P* > 0.05) difference among different groups in terms of the percentage of cancer cells at the S phase. In addition, there was no significant (*P* > 0.05) difference between the cell cycle diagram of the non-hibernating plasma-treated and control cells. These observations indicated that hibernating common carp plasma induced the G2/M cell cycle arrest in 4T1 breast cancer cells.

### Inhibitory effect of hibernating plasma on the cancer cells migration

**Figure 5:**
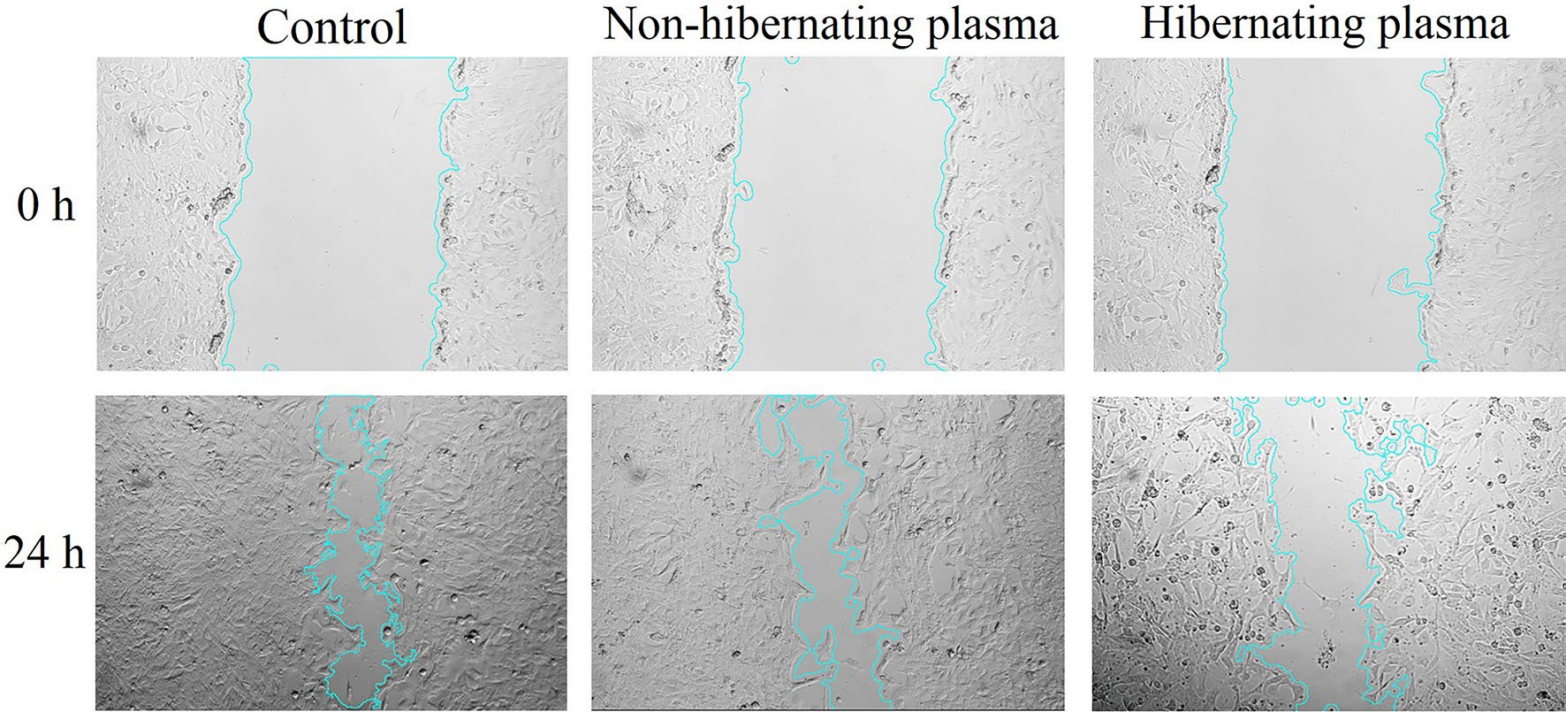
Effect of hibernating plasma on the migration of cancer cells according to the wound healing assay. Light microscope photographs of the scratched area immediately after wound formation (0 h) and after 24 h incubation with non-hibernating plasma (16 mg/ml) and hibernating plasma (16 mg/ml). The control wells were incubated with standard cell culture media. The wound area was quantitatively analyzed by the *Wound Healing Size Tool*, an ImageJ/Fiji1 plugin.

Finally, the anti-migration effect of hibernating plasma on 4T1 cancer cells compared to non-hibernating plasma was evaluated using the wound healing assay. As shown in Fig. 5, only 61.4 ± 6.1% of the wound area was closed in the hibernating plasma-treated wells (16 mg/ml) after 24 h, while this number was 83.3 ± 5.6% and 80.6 ± 4.4% for the non-hibernating plasma-treated and control wells, respectively. According to the wound closure percentage, no significant (*P* > 0.05) difference was observed between the control and non-hibernating plasma wells. Therefore, slower closure of the wound area after hibernating plasma treatment suggested a considerable (*P* < 0.05) anti-migration effect on breast cancer cells in this group compared to the non-hibernating plasma and control groups.

### In vivo inhibitory effects of hibernating plasma on the growth of 4T1 breast tumors

**Figure 6:**
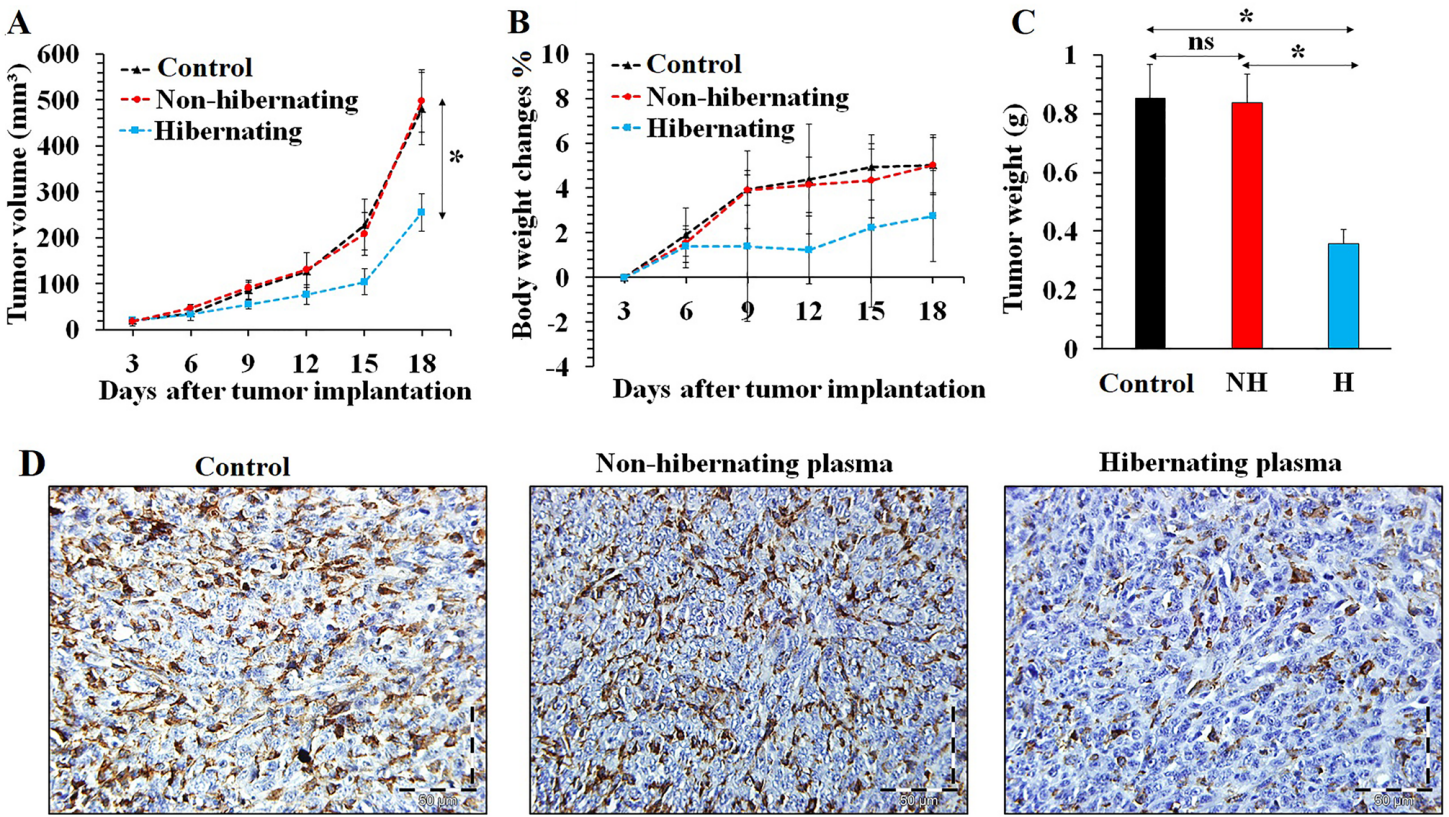
Evaluation of the anti-proliferative effect of hibernating plasma according to the tumor growth monitoring and the immunohistochemical analysis of the Ki-67 biomarker. (**A**) the mean tumor volume growth in the control (*n* = 5), non-hibernating plasma (*n* = 5), and hibernating plasma (*n* = 5) groups. (**B**) The mean body weight of tumor-bearing mice in different groups (*n* = 5). (**C**) The mean weight of tumors harvested on the 18th day after cancer cell implantation (*n* = 5). (**D**) Immunohistochemical detection of Ki-67 in breast tumors harvested from different groups (**n** = 5). (*NH* non-hibernating, *H* hibernating, *ns* non-significant. *: *P* < 0.05).

The tumor growth was inhibited by intratumoral injection of hibernating plasma compared with PBS (control) and non-hibernating plasma according to changes in the tumor volume progression (Fig. 6A) and body weight of 4T1 tumor-bearing mice (Fig. 6B). The tumor weight was significantly decreased in the hibernating plasma group in comparison with other groups (Fig. 6C). Based on our findings, the mean tumor volume and weight of the hibernating plasma-treated group (tumor volume: 255.1 ± 40.1 mm^3^, tumor weight: 0.35 ± 0.04 g) were significantly (*P* < 0.05) lower than the control group (tumor volume: 481.3 ± 68.3 mm^3^, tumor weight: 0.85 ± 0.11 g) on the last day of monitoring tumor growth. These anti-tumor effects were absent at the non-hibernating plasma-treated group (tumor volume: 497.9 ± 78.7 mm^3^, tumor weight: 0.83 ± 0.09 g) and no differences were detected between this group and the control group according to the mean tumor volume and weight growth. To confirm these anti-proliferative effects of hibernating plasma on 4T1 breast tumors, the analysis of Ki-67 as a biomarker of proliferating cells was carried out by immunohistochemical staining (Fig. 6D). The percentage of tumor cells that were positive for the Ki-67 biomarker was 20.5 ± 6.4%, 22.9 ± 7.3%, and 9.2 ± 5.6% for the control, non-hibernating plasma, and hibernating plasma groups, respectively. Therefore, the hibernating plasma-treated group exhibited a lower percentage of Ki-67-positive tumor cells compared with the control and non-hibernating plasma groups. The results of the immunohistochemical analysis were consistent with the results of tumor volume and weight growth associated with the anti-proliferative effects of hibernating plasma on 4T1 cancer cells in vivo.

### Anti-metastatic effect of hibernating plasma on prolongation of the breast tumor-bearing mice survival time

**Figure 7:**
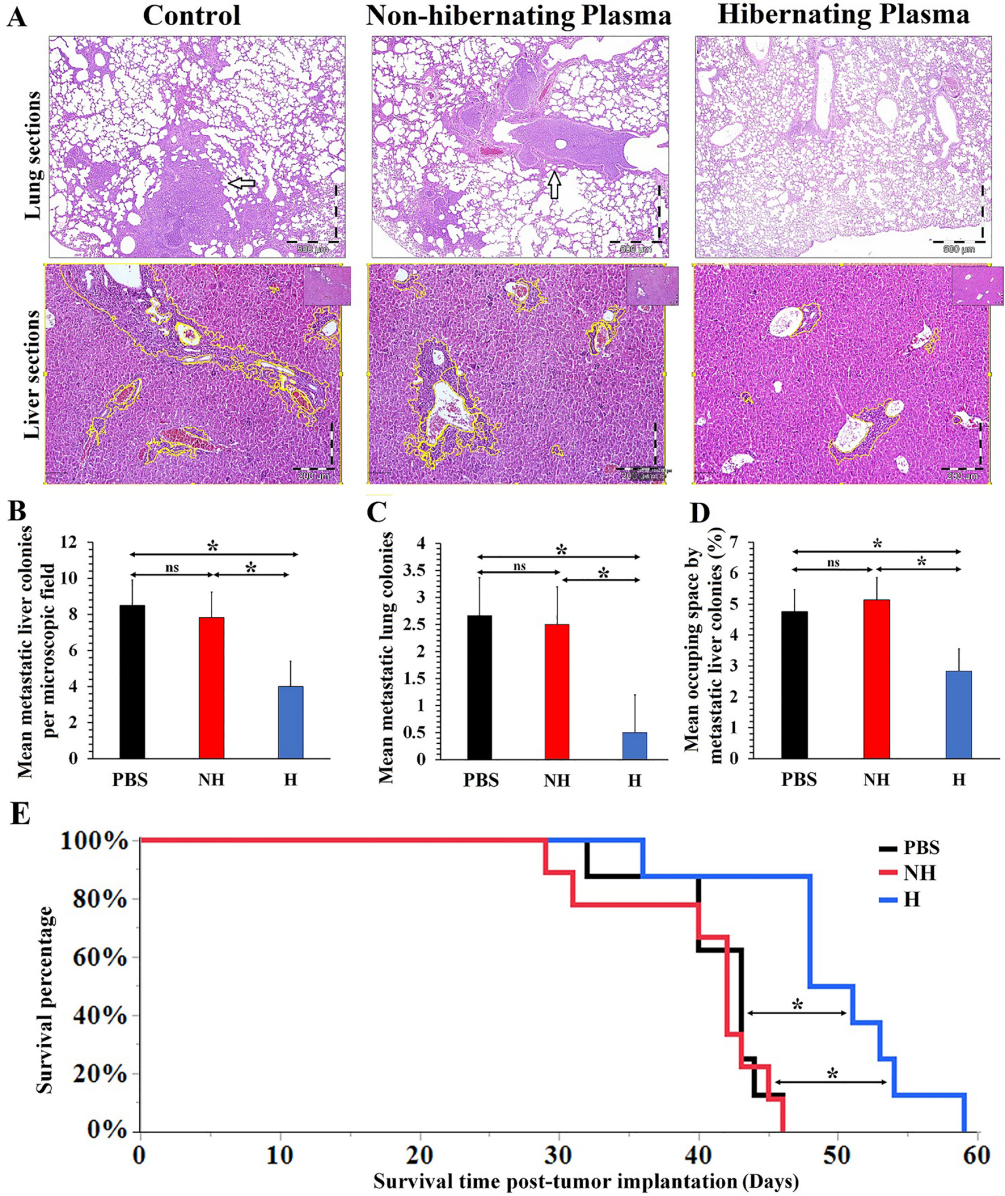
Effect of hibernating plasma treatment on the metastasis and survival time of tumor-bearing mice. (**A**) H&E-stained sections of the lungs and liver of tumor-bearing mice from different groups on the 40th day after cancer cells implantation (*n* = 5). The lungs metastatic colonies were pointed by an arrow. Also, the liver metastatic colonies were margined with the yellow lines by the Qupath software. (**B**) Mean number of metastatic colonies in the liver per microscopic field (Magnification × 100) according to H&E-stained sections analysis under a light microscope. (**C**) Mean number of metastatic colonies in the lungs according to H&E-stained sections analysis under a light microscope. (**D**) Mean occupying space by metastatic colonies in the liver per microscopic field (Magnification × 100) according to H&E-stained sections analysis under a light microscope. (**E**) The Kaplan–Meier survival curves of tumor-bearing mice in different groups (*n* = 8). (*NH* non-hibernating, *H* hibernating, *ns* non-significant. *: *P* < 0.05).

Therefore, the therapeutic effects of hibernating plasma injection on the formation of metastatic colonies in vital organs of tumor-bearing mice and their survival time were evaluated. On the 40th day after cancer cell implantation, the liver and lungs of mice were harvested to analyze metastasis burden (Fig. 7A). Significantly (*P* < 0.05) fewer metastatic colonies were observed in liver (Fig. 7B) and lungs (Fig. 7C) in the hibernating plasma-treated group (liver metastatic colonies: 4 ± 0.8, lungs metastatic colonies: 0.5 ± 0.5) compared with the control (liver metastatic colonies: 8.5 ± 1.2, lungs metastatic colonies: 2.6 ± 0.9) and non-hibernating plasma-treated groups (liver metastatic colonies: 7.8 ± 1.3, lungs metastatic colonies: 2.5 ± 0.5). Besides, the metastatic colonies significantly (*P* < 0.05) occupied lower space in the liver sections of the hibernating plasma-treated group in comparison with the other two groups (Fig. 7D). Furthermore, the hibernating plasma-treated mice had the longest mean survival time compared with mice in the other groups (Fig. 7E). A significant increase in the mean survival time of the hibernating plasma-treated tumor-bearing mice was observed compared to the control and non-hibernating-treated groups. These therapeutic effects were specific to hibernating plasma and were not observed for non-hibernating plasma.

### Analysis of plasma proteins by SDS-PAGE and identification with mass spectrometry (HPLC–ESI–MS)

**Figure 8:**
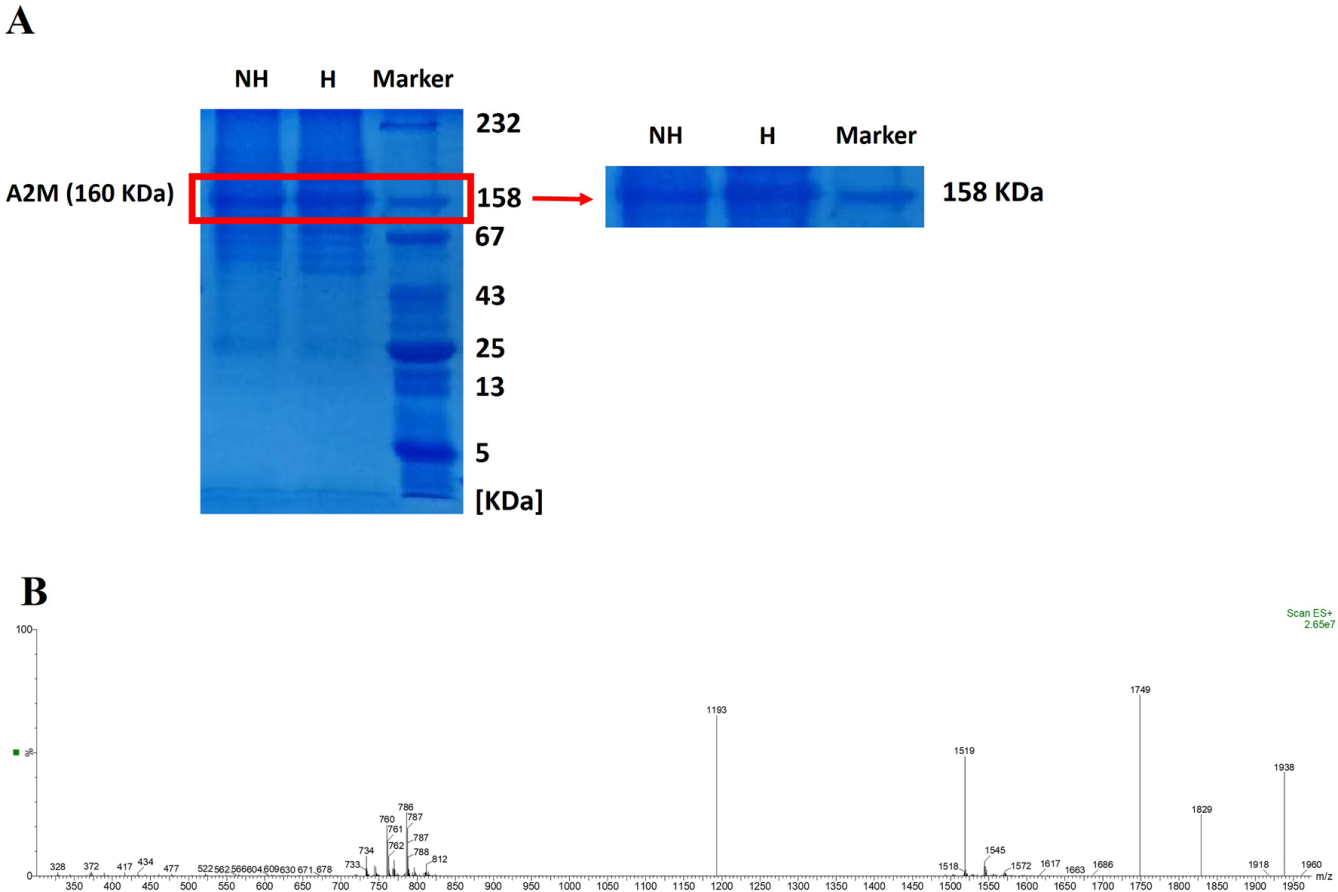
Analysis of plasma proteins by (**A**) SDS-PAGE (12%) and (**B**) identification with mass spectrometry (HPLC–ESI–MS). (Marker: Molecular weight marker of the proteins, NH: Non-hibernating *Common carp* plasma, H: Hibernating *Common carp* plasma, A2M: Alpha-2-Macroglobulin).

Protein concentration of the lyophilized plasma was 1.1 mg/ml. The gel electrophoresis of the plasma proteins of hibernating and non-hibernating common carp showed a large number of protein bands separated based on their molecular weights (Fig. 8A). The more robust protein bands were 158 kDa. The 158 kDa bands were related to alpha-2-macroglobulin, which has a molecular weight in this range. Based on our findings which calculated by Image J software, the thickness of this band in the hibernating common carp plasma was significantly 37.5 ± 10.3% (*P* < *0.05*) more than the non-hibernating common carp plasma.

The molecular mass of unmodified alpha-2-macroglobulin from common carp was determined 160.836 KDa (Uniprot: Q9PVU5_CYPCA). The results of the spectrum indicated the presence of alpha-2-macroglobulin (Fig. 8B). Meanwhile, the mass-to-charge ratio of this protein was quite consistent with alpha-2-macroglobulin. The charge-to-mass ratio of alpha-2-macroglobulin is as follows:

$$ \begin{aligned} \left( {{\text{M}} + {\text{ 83H}}} \right){83} + & = {1938}.{78} \\ \left( {{\text{M}} + {\text{ 88H}}} \right){88} + & = {1828}.{68} \\ \left( {{\text{M}} + {\text{ 92H}}} \right){92} + & = {1749}.{22} \\ \left( {{\text{M}} + { 1}0{\text{6H}}} \right){1}0{6} + & = {1518}.{32} \\ \left( {{\text{M}} + {\text{135H}}} \right){135} + & = \,{1192}.{38} \\ \end{aligned} $$

## Discussion

Hibernation is a state of minimal activity and metabolic depression. Previous studies have reported significant mitosis and cell cycle arrest in normal tissues during hibernation state. Several mechanisms were suggested to explain the suppression of mitosis in hibernating organisms. One of them is the catastrophic decrease in enzymatic activity due to the drop in the body’s core temperature to ambient temperature in which cells are unable to enter mitosis^27^. However, many studies have not supported this hypothesis and declared that the degree of metabolic suppression in animal models of hibernation cannot be explained by a decrease in temperature alone, and there must be a physiological inhibitory mechanism^28^. On the other hand, some studies have attributed these effects to plasma and its proteins^17–19,29^. Sieckmann et al*.* reported that plasma from hibernating 13-lined ground squirrels and woodchucks significantly suppressed murine splenocytes mitosis, while this inhibitory effect was not observed for the plasma of active and non-hibernating animals^24^. Their findings revealed that a protein with a large molecular weight that is alpha-2-macroglobulin may be responsible for this effect. Moreover, research done by Lyman et al*.* showed that the rate of proliferation of tumor cells that have been transplanted into hibernating hamsters was dramatically decreased^30^.

Despite the efforts made in this regard, our knowledge about hibernation in ectothermic organisms and the effect of hibernating ectotherms plasma on cancer cells is extremely limited. Most studies have been performed on hibernation models in mammals and reported the effects of these animal’s plasma on normal cells. Robbins et al*.* used the American bullfrog as a source of hibernating ectotherms plasma^13^. They reported an active suppression of mitosis and a significant cell cycle arrest in human THP-1 cells incubated with hibernating American bullfrog plasma, while non-hibernating plasma was unable to induce the same effects. Based on the findings from previous studies, we found that the plasma from hibernating animals may inhibit the proliferation of malignant cells. Thus, the main aim of this study was to assess the anti-cancer effects of hibernating common carp plasma on breast cancer cells in vitro and in vivo.

In this study, 4T1 mammary carcinoma, which is a highly tumorigenic, invasive, and metastatic murine triple-negative breast model, was selected to evaluate the anti-cancer effects of hibernating plasm. This model has several similarities with breast cancer in humans and is widely used in different studies^31^. Likewise, common carp (*Cyprinus carpio*) was selected to supply hibernating plasma. The common carp is one of the most globally distributed farmed fish that is cheap^25^. It is a eurythermal organism that can adjust to extreme seasonal variations in temperature from < 4 °C up to > 38 °C^32^. Previous studies demonstrated that lowering the temperature had a hibernation effect on cyprinoid fish such as crucian carp and common carp^26,33^.

Cancer treatments should cause the highest toxicity on tumor cells while exerting the lowest effects on normal cells^34^. The main weakness of current chemotherapy agents is their side effects because, in addition to cancer cells, they kill normal cells, which severely limits their use in the clinic^35^. Compared to chemical agents, natural products have shown low toxicity, high therapeutic efficacy, and better patients compliance^36,37^. Therefore, many studies have focused on natural products in identifying novel cancer treatments and hibernating plasma has the potential to become one of them. According to the results of this study, hibernating common carp plasma has significant anti-proliferative and pro-apoptotic effects on cancer cells, without any toxic effects on normal cells.

One of the hallmarks of cancer cells is continual uncontrolled cell proliferation^4^. Cell cycle control as a major regulatory mechanism of cell proliferation is one of the most effective strategies for cancer treatment by inhibiting the division of cancer cells. Many cytotoxic agents and/or DNA damaging agents have been used to arrest the cell cycle at the G0/G1, S, or G2/M phases^38^. In this study, hibernating plasma significantly increased the proportion of 4T1 cells in the G2/M phase and decreased in the G0/G1 phase. This was in line with previous studies that reported G2 phase arrest of enterocytes in the intestines of hibernating animals^18,19^. Moreover, plasma of hibernating American bullfrogs caused the arrest of human THP-1 cells at the G2/M phase^13^. Many studies have reported that arrest at the G2/M cell cycle phase causes apoptosis in different cancer cells including breast cancer cells^39–41^.

Meanwhile, a significant anti-proliferative effect was observed after intratumoral injection of the hibernating plasma to 4T1 breast tumors. Hibernating plasma injection inhibited the progression of tumor growth. Interestingly, significant inhibition of metastatic colony formation in vital organs was observed after the treatment with hibernating plasma, which significantly increased the survival time of tumor-bearing mice in this group. Altogether, hibernating plasma may include a variety of factors with anti-proliferative and anti-metastatic effects, although further analysis is needed to determine responsible factors for these traits.

In this regard, the electrophoretic pattern of the plasma proteins of hibernating and non-hibernating common carp demonstrated a thick band about 158 KDa. This band was related to alpha-2-macroglobulin (160 KDa), which has a molecular mass in this range. Moreover, mass spectrometry (HPLC–ESI–MS) confirmed that the molecular mass of this band and the unmodified alpha-2-macroglobulin (160.836 KDa) were completely consistent^42,43^. In agreement with previous studies^44,45^, the alpha-2-macroglobulin level was significantly increased in the hibernating common carp plasma compared to the non-hibernating common carp plasma in this study. Meanwhile, it has been highlighted that increased level of alpha-2-macroglobulin modulates tumour cell adhesion, migration, development and growth by inhibition of tumour promoting signalling pathways. Furthermore, it could inhibit tumour cell proliferation and the ability of cancers to infiltrate and metastasize and also induce apoptosis in cancer cells^46–49^. The unique properties of alpha-2-macroglobulin confirmed that the increased level of this protein in the plasma of hibernating common carp may have anti-cancer effects on breast cancer cells in this study.

## Conclusions

According to the results, the plasma of hibernating common carp significantly inhibited cell proliferation by inducing apoptosis and cell cycle arrest. Furthermore, hibernating plasma significantly reduced growth progression and metastasis of 4T1 breast tumors. Additionally, a significant increase in the mean survival time of the hibernating plasma-treated tumor-bearing mice was also observed. Taken together, our results indicated significant anti-proliferative and anti-metastatic effects of hibernating plasma on cancer cells that can be used as a new strategy to treat breast cancer. However, there may be different mechanisms underlying the anti-cancer effects of hibernating common carp plasma on breast cancer cells, our findings demonstrated the increased level of alpha-2-macroglobulin in plasma of this fish during hibernating could be a key factor.

## Material and methods

### Preparation of the fish for hibernation

All procedures involving animals were approved by the Animal Care and Use Committee of University of Isfahan (IR.UI.REC.1398.072). Alive adult common carps (mean weight: 1670 ± 240 g, mean length: 34.4 ± 7.9 cm) were purchased from a local fish farm in Isfahan provenance, Iran. They were placed in a 200-L plastic tank with circulating aerated water (Temperature: 20 °C, Flow rate: 500 L/h, Dissolved oxygen: > 5 mg/L) for one month. After acclimation period, the fish were randomly divided into two groups including non-hibernating and hibernating. Non-hibernating fish were kept in the same containers and conditions (Temperature: 20 °C). The hibernation protocol was done according to previous studies^13,26,50^. The fish were induced into a hibernated state by gradient cooling of water at the rate of 1–2 °C until 3 °C in water tanks located in a temperature-controlled cold room and maintained at this temperature during the winter. Then, we separately put the fish of each groups in a water tank containing eugenol (4-allyl-2 methoxy phenol, the active component of clove oil, 40 mg/L) to anesthetize them. Anesthesia of fish is characterized by the complete loss of balance, immobility, and failure to respond to strong external stimuli, such as intense touch and vibration of the tail area^51^. The fish were anesthetized by eugenol (40 mg/L) and blood was collected from the caudal vasculature. Subsequently, plasma was isolated, separately pooled together for each group, lyophilized and stored at -80° C for further use.

### Cell culture

The murine triple-negative mammary cancer (4T1) and normal murine fibroblast (L929) cell lines were obtained from the Pasteur Institute (Tehran, Iran). 4T1 and L929 cell lines were cultured in RPMI-1640 (Sigma-Aldrich, USA) supplemented with 10% fetal bovine serum (FBS; Sigma, USA) and 1% penicillin–streptomycin (Sigma-Aldrich, USA) and incubated at standard cell culture condition (37 °C, 5% CO2).

### MTT assay

The cell viability of 4T1 and L929 cells was evaluated using MTT (3-(4 5-dimethylthiazol-2-yl)-2 5-diphenyltetrazolium bromide) assay. Briefly, cells were allowed to grow to 80% confluency. The cells were detached using 0.25% (w/v) trypsin-ethylenediaminetetraacetic acid (EDTA) solution (GIBCO, USA) and seeded in 96-well culture plates at density of 5 × 10^3^ cells/well. After 24 h incubation, different concentrations (0, 1, 2, 4, 8, 16, 32 mg/ml) of lyophilized plasma of hibernating and non-hibernating common carp dissolved in culture medium were added to each wells. After 24, 48, and 72 h of cells seeding in 96 -well plate, cells were washed with phosphate-buffered saline (PBS), and then media were replaced with a basal culture medium containing 0.005% MTT solution (Sigma-Aldrich, USA). After 4 h incubation (37 °C, 5% CO2) in the dark, the medium was discarded and the precipitated formazan crystals were dissolved in dimethyl sulfoxide (DMSO). Finally, the absorbance of each well was detected by an Absorbance Microplate Reader (BioTek-ELX800, USA) at the wavelength of 570 nm. According to the following equation, the percentage of cell viability was calculated by relative value to the untreated cells (0 mg/ml) as the control group. The experiment was repeated three times and at least six wells were used for each concentration.

$$ Cell \,Viablity\, \left( \% \right) = \frac{{\left( {OD \,Sample - OD \,Blank} \right)}}{{\left( {OD \,Control - OD \,Blank} \right)}} \times {1}00 $$

### Morphological assessment

4T1 and L929 cells were separately seeded in 6-well culture plates (2.5 × 10^5^ cells/well) and incubated for 24 h. Then, the wells were washed three times with PBS (Sigma, USA). An inverted phase-contrast light microscope (Olympus, Japan) was used to capture photographs before treatment. Then, the wells were divided into three groups including control, hibernating plasma, and non-hibernating plasma. The untreated cells (0 mg/ml) were used as the control group, while the cells of hibernating plasma group were treated with the optimal hibernating plasma concentration selected from the MTT assay (16 mg/ml). The same concentration of non-hibernating plasma (16 mg/ml) was used to treat the cells of hibernating plasma group. At least, three wells were prepared for each group. After 24 h, the wells were washed three times with PBS and microscopic photographs were captured to evaluate changes in cellular morphology.

### Acridine orange/ethidium bromide (AO/EB) staining assay

The dual-fluorescent AO/EB staining assay was used to evaluate the cell viability as well as apoptosis. 4T1 cells were seeded in 6-well culture plates at a density of 1 × 10^5^ cells/well and incubated for 24 h. Subsequently, the cells were treated with the optimal hibernating and non-hibernating plasma concentration (16 mg/ml) for 24 h. The untreated cells (0 mg/ml) were used as the control group. At least, three wells were prepared for each group. Then, the wells were washed three times with PBS and stained with 100 µl AO/EB mixture solution containing 100 µg/ml acridine orange (Sigma-Aldrich, USA) and 100 µg/ml ethidium bromide (Sigma-Aldrich, USA) in PBS for 30 min in the dark. Finally, an inverted fluorescence microscope (Olympus, Japan) was used to capture images.

### Annexin V-fluorescein isothiocyanate (FITC) and propidium iodide (PI) staining assay

Apoptosis rate was determined by the apoptosis detection kit (BD Biosciences, USA) according to previous study^52^. 4T1 cells were seeded in 6-well culture plates at a concentration of 1 × 10^5^ and incubated overnight. Subsequently, the cells were treated with 16 mg/ml hibernating plasma or the same concentration of non-hibernating plasma for 24 h. The untreated cells (0 mg/ml) were used as the control group. The cells were detached and washed three times with cold PBS (1X, pH 7.4) and pelleted by centrifugation at 300 g for 5 min. Then, the cells were resuspended in binding buffer (100 µl), stained with 5 µl of Annexin V-FITC and the same volume of PI, and incubated for 15 min at room temperature in the dark. Subsequently, the volume of each sample was increased to 500 μl by adding binding buffer and the cells were immediately analyzed (10^4^ cells/sample) using flow cytometry (BD FACSCalibur, USA). This experiment was independently repeated three times and the obtained data were analyzed by the FlowJo-V10 software.

### Cell cycle analysis

The Tali™ cell cycle kit (Invitrogen/Life Technologies, USA) was performed to determine the percentage of cells at each stage of the cell cycle. 4T1 cells were seeded in 6-well culture plates at a density of 1 × 10^5^ and incubated overnight. Subsequently, the cells were treated with hibernating plasma (16 mg/ml) or non-hibernating plasma (16 mg/ml) for 24 h. The untreated cells (0 mg/ml) were used as the control group. After that, the cells were harvested by trypsinization and washed twice with cold PBS. Then, they were fixed with dropwise addition of 70% ice-cold ethanol (1 ml) while gentle vortexing and stored at -20 °C overnight. The fixed cells were washed twice with PBS and pelleted by centrifugation at 300 g for 5 min. Then, the cell pellet was resuspended in 200 µl of Tali™ cell cycle solution. Finally, it was incubated for 30 min at room temperature in the dark. The DNA content of stained cells was analyzed using flow cytometry (BD FACSCalibur, USA) for each sample. This experiment was independently repeated three times and the results were displayed as histograms. The obtained data were analyzed by the FlowJo-V10 software.

### Wound healing assay

The wound healing (or scratch) assay was carried out for cell migration analysis. 4T1 cells were seeded in 6-well culture plates and incubated for 48 h to grow to 80% confluency. Then, the cell layer was scratched by a sterile P200 Micropipette tip. The wells were washed twice with PBS to remove the debris and smoothing the edges of the scratch. Next, the cells were incubated with RPMI-1640 medium containing 0.5% FBS and treated with the optimal hibernating or non-hibernating plasma concentration (16 mg/ml) for 24 h. The untreated cells (0 mg/ml) were used as the control group. At least, three wells were prepared for each group. The photographs were obtained from the selected regions using an inverted phase-contrast light microscope (Olympus, Japan) at 0 h (immediately after media replacement) and 24 h after treatment with hibernating and non-hibernating plasma. The *Wound Healing Size Tool*, as an ImageJ/Fiji1 plugin, was used to quantifying the wound area^53^. The following equation was used to calculate the wound closure percentage.

$$ Wound\; closure\, \% = \left. {\left( {\frac{Initial\; wound\; area - Final\; wound\; area }{{Initail\; wound\; area}}} \right.} \right) \times 100 $$

### Cancer cells implantation and tumor challenge

All animal experiments and procedures were approved by the Animal Care and Use Committee of University of Isfahan (IR.UI.REC.1398.072). A total of 54 female Balb/c mice (mean weight: 23 ± 2 g) were purchased from the Royan Institute of Isfahan, Iran. The mice were housed in standard cages with controlled temperature (24 ± 2 ºC), relative humidity (50 ± 10%), 12-h light–dark cycle, and ad libitum access to enough food and water. It should be mentioned that standardized humane endpoints based on the current guidelines for endpoints in animal tumor studies were used^54–56^. After two weeks of acclimation, the mice's belly hair was shaved and disinfected with 70% alcohol. 1.5 × 10^6^ 4T1 cells suspended in 50 µl of basal culture medium were subcutaneously injected into the fourth left mammary fat pad of each mouse. When the tumor size reached 50–70 mm^3^, the mice were randomly divided into three groups including control, hibernating plasma, and non-hibernating plasma. The control group of mice was intratumorally injected with PBS (200 µl), while the other groups were respectively treated with intratumoral (i.t) injection of hibernating plasma (200 µl, 16 mg/ml) and non-hibernating plasma (200 µl, 16 mg/ml) once every day for 18 days. For tumor growth analysis, 15 Balb/c mice were injected with 4T1 cancer cells and randomly divided into the above-mentioned three groups (n = 5). The tumor volume was calculated using the following equation.

$$ Tumor\; volume = \frac{{\left( {Tumor\; length} \right) \times \left( {Tumor\; width} \right)^{2} }}{2} $$

The greatest longitudinal diameter (length) and the greatest transverse diameter (width) of tumors were measured by a digital caliper (Neiko, USA) every three days. Besides, the body weights of tumor-bearing mice were measured. For survival analysis, 24 Balb/c mice were injected with 4T1 cancer cells and randomly divided into the above-mentioned three groups (n = 8). The tumor-bearing mice were closely monitored for 60 days after tumor implantation. The animals’ death was recorded every day. The failure to eat and drink for over 3 days and a lack of limb movement were considered as the standardized humane endpoint used to euthanize animals by an overdose of ketamine/xylazine solution.

### Metastasis analysis

Metastasis is the main cause of death for cancer patients^57,58^. For metastasis analysis, 15 Balb/c mice were injected with 4T1 cancer cells and randomly divided into the above-mentioned three groups (n = 5). The tumor implantation and treatment approaches were as described in the previous section. The mice were sacrificed by overdose of ketamine/xylazine and their liver and lungs were harvested and fixed in 10% neutral buffered formalin solution 40 days after cancer cell implantation. An automatic tissue processor (Sakura, Japan) was employed to process the fixed samples. Then, a microtome (Leica Biosystems, Germany) was utilized to cut 4 µm thickness serial sections from the paraffin-embedded blocks. The sections were stained with Hematoxylin & Eosin (H&E) staining protocol for subsequent histological evaluation^59^. A minimum of 10 random microscopic fields was observed under the 10 × objective lens of a light microscope (Olympus, Japan) to report the mean number of metastatic colonies per microscopic field of the liver. Furthermore, the occupied area by metastatic colonies in each microscopic field of the liver (magnification × 100) was quantified by the Qupath software. The mean percentage of occupied space by liver metastatic colonies in each microscopic field was reported for each sample. Besides, all over the H&E stained sections of lungs were analyzed for counting metastatic colonies.

### Immunohistochemistry

Immunohistochemical staining of the proliferation marker Ki-67 was carried out to evaluate the anti-proliferative effect of hibernating plasma on 4T1 breast tumors. This biomarker is commonly used in the clinic for identifying the prognosis and effectiveness of treatment in patients^60,61^. For the analysis of Ki-67, 15 Balb/c mice were injected with 4T1 cancer cells and randomly divided into the above-mentioned three groups (*n* = 5). The tumor implantation and treatment approaches were as described in the previous section. The mice were sacrificed with the overdose of ketamine/xylazine solution after 18 days and the tumors were harvested. After 24 h fixation in 10% neutral buffered formalin, the specimens were processed by an automatic tissue processor (Sakura, Japan), followed by routine methods for subsequent histological evaluation. The sections were blocked with 5% bovine serum albumin and incubated with anti-Ki-67 antibody (Biolegend, USA) at 4 °C overnight. The sections were incubated with biotinylated anti-mouse IgG followed by incubation with streptavidin-labeled horseradish peroxidase (HRP). After three times wash, the sections were incubated with 3,3'-diaminobenzidine (DAB) tetrahydrochloride and followed by a counterstain with hematoxylin^62^. The photographs of slides were taken by using a digital light microscope (Olympus, Japan), and the percentage of tumor cells that were positive for the Ki-67 as a proliferation biomarker was quantified using ImageJ software^63^.

### Analysis of plasma proteins by SDS-PAGE and mass spectrometry (HPLC–ESI–MS)

Sodium Dodecyl Sulfate–Polyacrylamide Gel Electrophoresis (SDS-PAGE) was carried out to analyze the plasma proteins of hibernating and non-hibernating common carp. Firstly, 1 mg of the lyophilized plasma of hibernating and non-hibernating common carp was separately dissolved in 1 ml PBS buffer. Protein concentration of the lyophilized plasma was determined using a microplate reader (BioTek-Synergy HT, USA). Then, 15 µl of dissolved lyophilized plasma was mixed with 5 µl of sample loadings buffer for SDS-PAGE (1X; Sigma-Aldrich, USA) and heated at 100 °C for 10 min. After that, 20 µl of this solution was loaded into each well of the gel (12% SDS-PAGE). The gels were electrophoresed at 70 V for 30 min and then at 130 V for 1.5 h using a SDS gel running buffer. The molecular weight marker of the protein (ranging from 5–232 KDa) included Insulin (5 KDa), Ribonuclease A (13 KDa), Chymotrypsinogen A (25 KDa), Ovalbumin (43 KDa), Bovin Serum Albumin (67 KDa), Aldolase (158 KDa), Catalase (232 KDa) was loaded in a lane next to the plasma samples. The gels were stained with Coomassie Blue dye (Sigma-Aldrich, USA) for 45 min at 25 °C after electrophoresis. Hot water and a shaker (IKA-Werke, Germany) were used to decolorize the gel. Finally, the gels were imaged using a Bio-5000 Plus gel imaging scanner (Zhongjing Microtek, Shanghai, China), and the density of protein bands was measured by Image J Software. The 158 KDa band cut off from the gel and send for the high-performance liquid chromatography/electrospray ionization tandem mass spectrometry (HPLC–ESI–MS) analysis performed by Waters Alliance 2695 HPLC-Micromass Quattro micro API Mass Spectrometer.

### Statistical analysis

The data were expressed as the mean ± standard deviation (SD). One-way ANOVA and Tukey’s Post Hoc tests were used to evaluate the significance of the difference between groups, by employing the JMP 14.0 software (SAS Institute, Japan). The difference was considered statistically significant if *P* < 0.05. (*: *P* < 0.05, ns: not significant). The Kaplan–Meier curves were drawn and analyzed by JMP software, using the Log-Rank test to assess the significant differences between the mean survival time of tumor-bearing mice in different groups.

### Ethical approval

All animal experiments and procedures were conducted according to the Guidelines for the Care and Use of Laboratory Animals of the University of Isfahan, which refer to the American Association for Laboratory Animals Science and the guidelines laid down by the NIH (NIH Guide for the Care and Use of Laboratory Animals) and also ARRIVE guidelines. All experimental protocols were approved by the Ethics Committee of the University of Isfahan, Iran (IR.UI.REC.1398.072).

## Data Availability

All datasets used and/or analysed during the current study are available from the corresponding author on reasonable request.
